# P-1599. XBB.1.5 responses present up to a year post COVID-19 mRNA vaccination in long-term care residents with a strong boost from KP.2 vaccination

**DOI:** 10.1093/ofid/ofaf695.1778

**Published:** 2026-01-11

**Authors:** David Canaday, Olajide Olagunju, Clare Nugent, Laurel Holland, Evan Dickerson, Tiffany Wallave, Yasin Abul, Ellen See, Mike Payne, Jurgen Bosch, Chia Jung Li, Eunice Lim, Ivis Perez, H Edward Davidson, Lisa Han, Christopher King, Alejandro Balazs, Brigid WIlson, Stefan Gravenstein

**Affiliations:** VA Northeast Ohio Healthcare System, Cleveland, OH; Case Western Reserve University, Cleveland, Ohio; Brown, Providance, Rhode Island; Rhode Island Hospital, Providence, Rhode Island; Rhode Island Hospital, Providence, Rhode Island; Rhode Island Hospital, Providence, Rhode Island; Brown University, Providence, Rhode Island; Case Western Reserve University, Cleveland, Ohio; CWRU, Cleveland, Ohio; CWRU, Cleveland, Ohio; Harvard University, Boston, Massachusetts; Harvard University, Boston, Massachusetts; Insight Theraputics, Norfolk, Virginia; Insight Therapeutics LLC, Norfolk, Virginia; Insight Therapeutics, LLC, Norfolk, Virginia; Case Western Reserve University, Cleveland, Ohio; Harvard University, Boston, Massachusetts; Cleveland VA, Cleveland, Ohio; Brown University, Providence, Rhode Island

## Abstract

**Background:**

While CoVID-19 vaccination dramatically reduced hospitalization and mortality, still 10% of nursing home residents (NHRs) with COVID-19 get hospitalized. This is partly due to failure to be up-to-date in vaccination, emerging variants, and waning immunity. Despite ACIP’s twice-yearly vaccination recommendation for NHR, mid-season vaccine uptake remains low. This study assesses residual immunity after 1 or 2 2023-24 XBB.1.5 mRNA vaccine doses and the 2024-25 KP.2 vaccine.

**Fig. 1.**
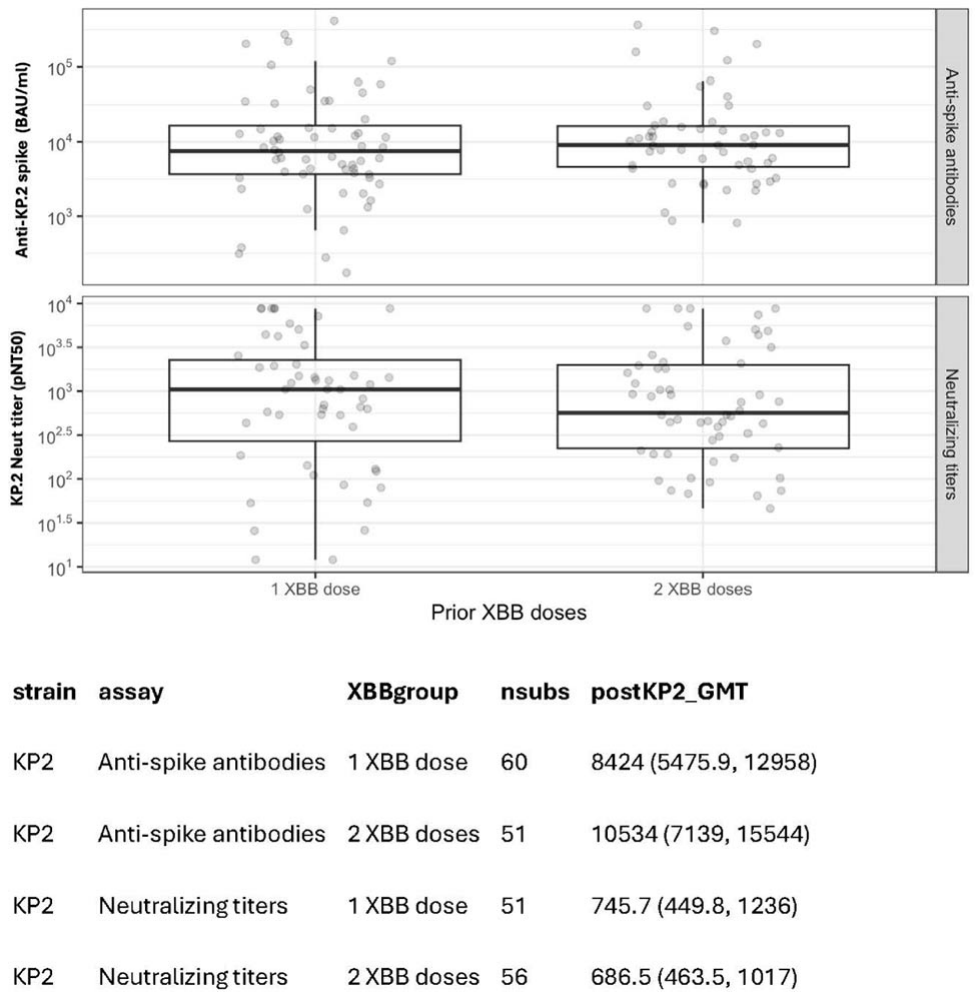
Post KP.2 vaccination anti-KP.2 spike and neutralization titers in NHR with 1 or 2 prior XBB.1.5 vaccines. Geometric mean titers were compared using t-tests on log-transformed values.

**Fig. 2:**
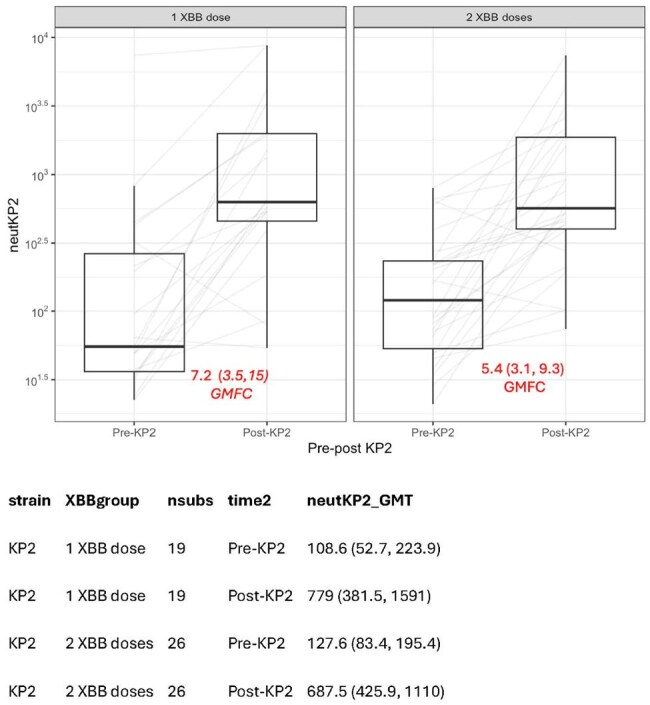
Fold rise of KP.2 neutralization titers pre- and post-KP.2 vaccine in NHR with history of 1 or 2 prior XBB.1.5 vaccines. Geometric fold change (GMCK) shown in red. Geometric mean titers and fold change were compared using t-tests on log-transformed values.

**Methods:**

The NHRs were part of two longitudinal cohort studies in OH and RI examining COVID-19 vaccine immune response in 116 long-stay NHR. We assessed vaccine-induced XBB.1.5 and KP.2 response using bead-multiplex immunoassay for anti-spike and pseudovirus neutralization assay (50% Pseudovirus Neutralizing Antibody Titers (pNT50)). Among subjects with samples obtained in the 60 days prior to KP.2 vaccine, we assessed XBB.1.5 titer levels before and KP.2 titer fold change following vaccination.

**Results:**

In this study, post-KP.2 samples from 129 NHR were studied, of whom 45 had pre-KP.2 vaccine samples available. The vast majority of NHR had prior infection, and all subjects had received multiple prior vaccine doses. The post-vaccine levels of both KP.2 pNT50 and anti-KP.2 spike antibody were similar across prior XBB.1.5 doses (Fig. 1, p=0.80 and p = 0.44). Among those with both pre- and post-KP.2 samples, NHR with a single XBB.1.5 vaccination had GMT(95% CI) XBB.1.5 pNT50 titers of 994 (540, 1829) at median (IQR) 298 (288, 314) days post-vaccination; those with two XBB.1.5 vaccinations had GMT (95% CI) XBB.1.5 pNT50 of 1338 (661, 2710) at 131 (127,134) days after their second vaccination. The magnitude of KP.2 vaccination boosting of KP.2 neutralizing titers in NHR was similar whether a NHR had one or two prior XBB.1.5 boosters, with geometric mean fold changes 7.2 and 5.4, respectively (Fig. 2, p = 0.5).

**Conclusion:**

While anti-XBB.1.5 pNT50 levels in NHRs at 298 days post-vaccine suggest significant protective activity, they may not be for emerging variants. The substantial decline in protection over this time can be boosted in NHRs by mid-season vaccination. Meanwhile, NHR KP.2 vaccination generated robust responses independent of the number of prior XBB.1.5 doses were received.

Funder CDC

**Disclosures:**

David Canaday, MD, Moderna: Grant/Research Support|Pfizer: Grant/Research Support|Seqirus: Advisor/Consultant|Seqirus: Grant/Research Support|Seqirus: Honoraria Yasin Abul, MD, CDC/ABT: Grant/Research Support|CLARIO: Advisor/Consultant|GSK: Grant/Research Support|Moderna: Grant/Research Support|Seqirus: Grant/Research Support Ivis Perez, MPH, LPN, GSK: Grant/Research Support|Moderna: Grant/Research Support|Sumitomo: Grant/Research Support H Edward Davidson, PharmD, GSK: Grant/Research Support|Moderna: Grant/Research Support|Sumitomo: Grant/Research Support Lisa Han, MPH, GSK: Grant/Research Support|Moderna: Grant/Research Support|Sumitomo: Grant/Research Support Stefan Gravenstein, MD, MPH, GSK: Advisor/Consultant|GSK: Grant/Research Support|GSK: Honoraria|Moderna: Grant/Research Support|Novavax: Advisor/Consultant|Novavax: Honoraria|Pfizer: Advisor/Consultant|Pfizer: Grant/Research Support|Pfizer: Honoraria|Sanofi: Advisor/Consultant|Sanofi: Grant/Research Support|Sanofi: Honoraria|Seqirus: Grant/Research Support

